# Pathways linking health literacy to diabetes risk scores in a non-diabetic population in Ismailia, Egypt: a cross sectional study design

**DOI:** 10.1186/s12889-025-23526-1

**Published:** 2025-06-25

**Authors:** Mirella Youssef Tawfik, Rehab A. Mohamed, Noha M. Abu Bakr Elsaid

**Affiliations:** 1 Department of Public Health, Occupational and Environmental Medicine, Faculty of Medicine, Suez Canal University, Ismailia, Egypt; 2 Department of Family Medicine, Faculty of Medicine, Suez Canal University, Ismailia, Egypt

**Keywords:** Type 2 diabetes mellitus, Health literacy, Self-Efficacy, Preventive health behavior, ARABRISK, Path analysis

## Abstract

**Background:**

Type 2 diabetes mellitus (T2DM) is a growing public health issue in Egypt. Health literacy (HL) is a modifiable factor influencing diabetes risk (DR), but the pathways through which HL impacts diabetes risk remain unclear. This study aimed to examine the direct and indirect pathways linking HL to DR in a non-diabetic population.

**Methods:**

A cross-sectional study was conducted from July 2022 to August 2023 among employees in the administrative sectors of 16 faculties at Suez Canal University, Ismailia, Egypt. Each faculty was treated as a cluster for sampling. Eligible participants were those without a diagnosis of T2DM. Exclusion criteria included use of antidiabetic medications, cancer diagnosis, long-term corticosteroid or immunosuppressant use, or pregnancy. Sample size from each sector was determined proportionally based on the number of eligible employees, and participants were randomly selected from a coded list. Data were collected via structured face-to-face interviews using validated tools to assess HL, self-efficacy (SE), diabetes knowledge (DK), preventive health behaviors (PHB), and DR, measured by the ARABRISK score. Statistical analyses included Spearman correlations, non-parametric tests, and Structural Equation Modeling (SEM) via SAS PROC CALIS to assess direct and indirect pathways from HL to DR, controlling for significant covariates.

**Results:**

Of the participants, 59.4% had inadequate/problematic HL, and 54.8% had moderate-to-high DR. HL was positively correlated with DK (*r* = 0.275), SE (*r* = 0.379), and PHB (*r* = 0.514) and negatively correlated with DR (*r*=–0.542), all with p-values < 0.001. The strongest negative correlation was between PHB and DR (*r*=–0.957). SEM revealed a weak but significant direct effect of HL on DR (β= − 0.05108, *p* < 0.001). The most substantial indirect effect was observed through PHB (β= − 0.93663, *p* < 0.001). Additional indirect pathways through DK and SE also emerged, although SE had no significant effect on PHB.

**Conclusions:**

HL reduces DR primarily through its effect on PHB. While DK and SE contribute, their effects are less pronounced. Interventions that enhance HL and support healthy behaviors may help prevent T2DM in at-risk populations. Future research should use longitudinal designs, diabetes-specific HL tools, objective risk measures; explore psychosocial mediators; and study diverse populations.

**Supplementary Information:**

The online version contains supplementary material available at 10.1186/s12889-025-23526-1.

## Background

Diabetes Mellitus (DM) is one of the fastest-growing global public health emergencies of the 21st century. The Middle East and North Africa (MENA) region has the highest comparative prevalence (CP) of DM (18.1%) in people aged 20–79 years [1]. Egypt ranks second in the MENA region, with a CP of 20.9% in 2021, projected to rise to 23.5% by 2045 [2]. Approximately 95% of DM cases are type 2 DM (T2DM), which develops from a combination of modifiable and non-modifiable risk factors. Since symptoms of T2DM are often mild or absent, individuals may remain undiagnosed for years [3]. To facilitate early identification, the American Diabetes Association (ADA) and the International Diabetes Federation (IDF) [4, 5] recommend using diabetes risk assessment tools to screen for future T2DM risk in asymptomatic adults.

Health literacy (HL) is a potentially modifiable risk factor for T2DM [6]. It refers to an individual’s achieving a level of knowledge, personal skills, and confidence to make informed decisions that improve personal and community health [7]. Several studies have identified an inverse association between HL and the risk of developing T2DM [6, 8, 9], but the mechanisms linking HL to diabetes risk (DR) are not well understood.

Theoretical models have proposed possible pathways to explain the relationship between HL and health outcomes. Baker’s model of HL suggests that HL improves health outcomes through facilitating the acquisition of new knowledge, enhancing self-efficacy (SE), and promoting positive health behaviors (HB) [10]. However, this model does not explicitly outline the pathways through which these variables interact to influence health outcomes. Similarly, the Paasche-Orlow and Wolf model [11] conceptualizes HL as a predictor of knowledge and SE, both of which serve as key determinants of HB, a pathway through which HL influences health outcomes. However, these models do not propose a direct link between HL and health outcomes.

Beyond these theoretical models, empirical evidence supports the presence of internal pathway links between HL and health outcomes in populations at risk of developing T2DM. For example, HL has been positively associated with HB among adults with prediabetes [12], and adopting healthy behaviors has been shown to prevent progression to T2DM in individuals with impaired glucose tolerance or dysmetabolism [13].

Despite growing recognition of HL’s role in diabetes prevention, most research to date has focused on individuals already diagnosed with T2DM [14–16], while at-risk, non-diabetic populations remain underexplored. One study examining pathways in patients with T2DM demonstrated that SE and self-care behaviors mediate the relationship between HL and glycemic control, with SE facilitating engagement in self-care behaviors [14]. Other studies have emphasized the substantial role of HL in diabetes knowledge (DK) [15] and the mediating effect of DK in linking HL to reduced smoking as a self-management behavior [16]. Collectively, these studies underscore the relevance of Baker’s HL constructs—SE, DK, and health behaviors—as key mediators in the relationship between HL and diabetes-related outcomes.

Moreover, studies examining both the direct and indirect pathways linking HL to DR using comprehensive models and regionally adapted tools are scarce in countries from the MENA region, where HL levels are generally low and diabetes prevalence is high. Research in this context can inform targeted strategies for T2DM prevention before the onset of disease. To address this gap, the present study investigates how HL impacts T2DM risk in a sample of the non-diabetic population by validating a model that explores both direct and indirect pathways linking HL to T2DM risk scores. The model evaluates the direct effect of HL on diabetes risk (DR) and explores three indirect pathways: one through DK and preventive health behavior (PHB), another through SE and PHB, and a third through PHB alone.

## Methods

### Study design, setting, and participants

This study used a cross-sectional design and was conducted in the administrative sectors of the 16 faculties affiliated with Suez Canal University in Ismailia, Egypt, from July 2022 to August 2023. Each faculty was treated as a cluster for sampling. All employees who had not been diagnosed with T2DM were eligible for recruitment. Individuals were excluded if they were currently taking antidiabetic medications (regardless of diabetes diagnosis), diagnosed with cancer, receiving long-term corticosteroids or immunosuppressants, or pregnant at the time of recruitment. Participants were invited to join the study and provided with an explanation of the study objectives and procedures. Data collection took place during employees’ working hours, with careful attention paid to minimizing disruption to their work responsibilities. Participants were given the option to complete the questionnaire during scheduled breaks or at designated times that would not interfere with their job duties.

### Measures and tools

- Self-reported data were collected using a structured face-to-face interview questionnaire covering five key constructs: HL, SE for healthy foods and physical activity, DK, T2DM PHB, and DR.
- Health literacy was assessed using the validated Arabic 16-item short version of the European Consortium for Health Literacy Questionnaire (HLQ-EU-16) [17]. It is a self-reported, non-specific (general) tool that focuses on communicative and critical HL [18]. This tool consists of 16 items rated on a 4-point Likert scale, distributed across three subscales: health care (7 items), disease prevention (5 items), and health promotion (4 items). Response options range from 1 (very difficult) to 4 (very easy) and were dichotomized into 0 (very difficult/fairly difficult) and 1 (fairly easy/very easy) for scoring (with a range from 0 to16). Scoring establishes three HL categories: 0–8 = inadequate, 9–12 = problematic, and 13–16 = sufficient HL. This questionnaire has demonstrated high internal consistency reliability and construct validity [17].
- Self-efficacy was measured using the adapted Arabic version of the SE for Healthy Foods and Physical Activity Questionnaire (SEHFPA-Q) [19]. This questionnaire assesses key constructs influencing behavior, particularly SE beliefs related to choosing healthy foods and engaging in physical activity [20, 21]. The SEHFPA-Q consists of 19 items divided into two domains: SE for healthy eating (11 items) and SE for physical activity (8 items). The healthy eating domain includes three subscales: increasing fiber intake (3 items), decreasing fat intake (6 items), and reducing sugar consumption (2 items). The physical activity domain consists of two subscales: integrating physical activity into daily routines (6 items) and overcoming barriers to physical activity (2 items). Participants rated their confidence on a scale from 0 to 100, where 0 indicates “certainly I cannot,” 50 indicates “somewhat I can,” and 100 indicates “certainly I can.” For interpretation, self-efficacy scores were categorized as follows: low (0–33), moderate (34–66), and high (67–100) self-efficacy. This questionnaire demonstrated moderate to high internal consistency reliability, with Cronbach’s alpha ranging from 0.68 to 0.90 across subscales, and strong construct validity, with factor loadings between 0.65 and 0.96 [19]. Since the adapted questionnaire was initially tested only on females, a pilot study was conducted on a separate sample of 50 males and females to assess its reliability across genders. The results showed moderate to high internal consistency reliability, with Cronbach’s alpha ranging from 0.74 to 0.92.
- The DK and PHB were assessed by questionnaires developed by the authors. The DK Questionnaire was designed based on established guidelines from the IDF [22] and the ADA [23], while the PHB questionnaire was formulated following recommendations from the World Health Organization (WHO) [3]. A thorough review of existing validated questionnaires revealed that none fully aligned with the specific objectives of this study, particularly in capturing DK and PHB in a non-diabetic population. Therefore, the development of new instruments was necessary to ensure comprehensive and contextually relevant assessment. The DK questionnaire assesses participants’ knowledge across four domains: risk factors (10 items), symptoms (9 items), complications (6 items), and prevention (9 items). Each domain consists of a multiple-response question allowing participants to select all applicable answers. The total score (0–34) was calculated by summing correct responses, with higher scores indicating greater DK. For interpretive purposes, DK scores were categorized as follows: low knowledge (0–11), moderate knowledge (12–22), and high knowledge (23–34).
- The PHB questionnaire consists of 12 items assessing adherence to key diabetes prevention behaviors over the past three months. It is divided into four subscales: healthy eating (3 items), weight management (3 items), physical activity (3 items), and smoking cessation/prevention (3 items). Responses for the first three subscales were recorded on a 5-point Likert scale (0–4), corresponding to different frequencies (e.g., “never” to “usually” or “<10 minutes” to “>30 minutes”), resulting in subscale scores ranging from 0 to 12 and a total of 0 to 36 for these three domains. For the smoking cessation/prevention subscale, a conditional scoring system was used to differentiate between non-smokers and smokers. Non-smoking status was determined by the question, “Have you ever smoked?” If the response was “No,” no further smoking-related questions were asked, and the participant received the maximum subscale score of 12, equivalent to the highest possible score for a non-smoker. If the response was “Yes,” the participant was asked, “Have you attempted to quit smoking?” If the response was “No,” no further question was asked, and the participant received the minimum subscale score of 0, representing a current smoker. If the response was “Yes,” the participant received a subscale score of 4 and was asked a third question: “Are you still quitting now?” If the response was “No,” the participant’s subscale score remained at 4. If the response was “Yes,” the participant received an additional score of 4, resulting in a total subscale score of 8, and was classified as a former smoker. The total PHB questionnaire score was computed by summing all subscale scores, ranging from 0 to 48, with higher scores indicating greater adherence to PHB. A pilot study was conducted on 50 eligible employees (not included in the main study) to assess the questionnaire’s internal consistency. The Cronbach’s alpha for the first three subscales demonstrated excellent reliability (0.83 for physical activity, 0.89 for healthy eating, and 0.94 for weight management). Reliability testing was not conducted for the smoking prevention subscale, as smoking behavior is inherently variable over time. Additionally, internal consistency measures were not applicable due to the conditional structure of the subscale, where only current smokers responded to the final two questions. Content validity of the developed questionnaires was established through expert review by professionals in diabetes research and public health before use. PHB scores were categorized as low (0–15), moderate (16–31), and high (32–48).
- The validated ARABRISK tool [24] was used to assess DR in the study participants. The self-reported components of ARABRISK provided data on four demographic variables (age, gender, education level, and ethnicity), two health behavior variables (physical activity and healthy eating), and four medical history variables (hypertension, high blood glucose, high birth weight babies, if applicable, and family history of diabetes). Additionally, marital status and average monthly income were assessed separately, not as part of the ARABRISK score, but to facilitate further analysis of covariates associated with mediators and the dependent variable in subsequent statistical modeling. The clinical components of the ARABRISK tool included Body Mass Index (BMI) and waist circumference (WC). BMI was calculated as weight in kilograms (kg) divided by height in meters squared (m²), with values ≥ 25 kg/m² classified as overweight and ≥ 30 kg/m² as obese. Weight was measured to the nearest 0.5 kg and height to the nearest 1 cm, with participants standing upright and wearing minimal clothing. Waist circumference was measured to the nearest 0.5 cm at the midpoint between the lowest rib and the iliac crest at the end of normal expiration. Values of ≥ 80 cm in women and ≥ 94 cm in men were considered indicative of an increased risk of cardiovascular disease, metabolic syndrome, and type 2 diabetes mellitus (T2DM) [25], while values of ≥ 88 cm in women and ≥ 102 cm in men indicated a highly increased risk for these conditions [26].

Each item within the ARABRISK components was assigned an established score based on participant responses or measurement values, with higher scores indicating greater risk. The total ARABRISK score was calculated by combining the scores obtained from the self-reported and clinical components. Based on the total ARABRISK score, participants were classified into three risk categories: low risk (score < 21), moderate risk (score 21–32), and high risk (score ≥ 33). The ARABRISK tool has demonstrated high reliability and validity in Arabic populations [24].

### Sampling and sample size justification

The required sample size for this study was determined based on the number of predictors, mediator variables, and the desired power and significance level. Using the general formula for estimating the required sample size for mediation models [27], the calculation was performed as follows:

$$\eqalign{& N = [{t^2} \times ({n_{(predictors + )}}{n_m}ediators) \times (1 - {R^2}) \times (1 + {n_m}ediators)] \cr & \,\,\,\,\,\,\,\,\, \div [{R^2} \times (1 - {R^2}) + ({n_{(predictors + )}}{n_m}ediators) \times {R^2}] \cr} $$

Where: N is the required sample size, *t*= 4, corresponding to a power of 0.99 and a significance level of 0.01, *n*_*predictors*_ =1 (health literacy), *n*_*mediators*_ = 3 (DK, SE, and HB), R^2^=0.2 (the estimated proportion of variance explained by the predictor excluding mediators).

Based on this formula, the initial estimated sample size was 213 participants. However, an adjustment for the design effect (DEFF) was necessary due to the cluster sampling design. With 16 faculties as clusters and an average of 50 participants per faculty, the adjusted sample size was calculated using the formula [28]:

$$\:{N}_{adjusted=\:\:\:\:\:}{N}_{calculated\:\times\:DEFF}$$

**Fig. 1:**
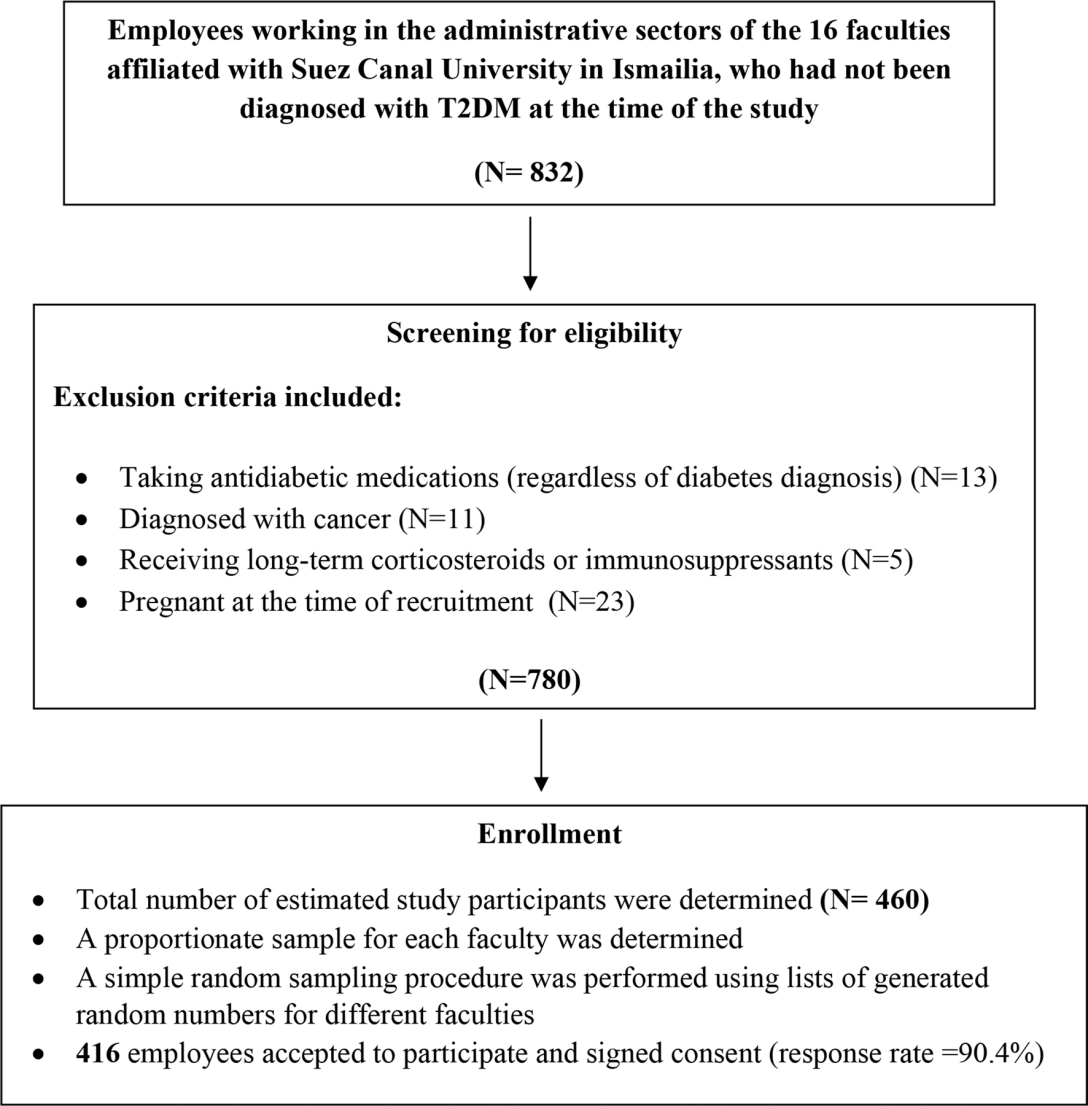
Flow chart of study participant

Where: *DEFF* (accounts for the fact that individuals within the same faculty (cluster)may share similar characteristics)=1+ (*m*-1)×ICC, with *m* = 50 (average cluster size) and an assumed intraclass correlation coefficient (ICC) of 0.018 [29]. This yielded an adjusted sample size of approximately 400 participants, which was further increased to 460 to allow for an expected non-response rate of 15%. The participant sample size from each faculty was determined based on the relative representation of eligible personnel within each sector. Using a random integer generator from a trustworthy software package (RANDOM.ORG’s integer generator), employees were randomly selected from a coded list of eligible participants and invited to participate in the study. All participants received a detailed explanation of the study’s objectives and methods. Those who accepted to participate signed consent forms, resulting in a final total of 416 participants and a response rate of 90.4% (Fig. 1 represents a flow chart of study participants).

### Data management and statistical analysis

SPSS version 27.0 was used to conduct the statistical analysis (IBM Corporation, Armonk, NY, USA). Since continuous data was found to be non-normal by the Kolmogorov-Smirnov test, medians and interquartile ranges (IQR) were utilized to display the data. Frequencies and percentages were used to display categorical variables. Correlation analysis between selected variables included in the path model was conducted using Spearman’s rank correlation coefficient. Additionally, the Mann-Whitney U test, Kruskal-Wallis test, and Spearman’s rank correlation were used to identify significant associations between covariates and HL, mediators (DK, SE, PHB), and DR. Correlation coefficients were interpreted as follows: ≥0.9 as very strong, 0.7–0.8 as strong, 0.4–0.6 as moderate, 0.1–0.3 as poor, and 0.0 as none [30].

Significant covariates were subsequently controlled for in the SEM model. Path analysis was performed using Structural Equation Modeling (SEM) implemented through the SAS PROC CALIS procedure. Structural Equation Modeling (SEM) was employed to evaluate both direct and indirect mediation effects among the study variables, testing the hypothesized pathways linking HL to Diabetes Risk DR. Model fit was assessed using multiple fit indices: Goodness of Fit Index (GFI), Normed Fit Index (NFI), Comparative Fit Index (CFI), Root Mean Square Error of Approximation (RMSEA), and Standardized Root Mean Square Residual (SRMR). The following thresholds were used for model evaluation: GFI and NFI ≥ 0.90 indicate an acceptable fit, CFI ≥ 0.95 represents a good fit, SRMR ≤ 0.08 is considered acceptable, and RMSEA ≤ 0.06 indicates a close fit, with values up to 0.08 suggesting reasonable approximation [31]. Indirect mediated effects were tested using bias-corrected bootstrapping with 5000 resamples, generating 95% confidence intervals (CI) to determine statistical significance. Standardized path coefficients (β) were reported to assess effect sizes, with values interpreted based on Cohen’s conventional thresholds, where (β) values of 0.10, 0.30, and 0.50 represent small, medium, and large effect sizes, respectively [32]. A p-value of < 0.05 was considered statistically significant for all analyses.

## Results

This study included 416 participants. The median age of participants was 41 years (IQR = 13), with only 7.9% in the youngest age group (20 - <30 years). The male-to-female ratio was approximately 2:3 (39.2% vs. 60.8%). Most participants (82.7%) had completed high school/diploma or less, and 20% had an average monthly income > 10,000 LE. Only 6% were unmarried. The majority (78.4%) had a BMI ≥ 25, placing most participants in the overweight/obese group, with a median of 28 (IQR = 5.4). Among females, 71.5% had a WC ≥ 80 cm, while among males, 53.4% had a WC ≥ 94 cm placing a substantial proportion of participants in the increased risk/highly increased risk group, with a median WC of 84 cm (IQR = 13) for females and 94 cm (IQR = 14) for males. A total of 25.5% of participants engaged in at least 30 min of daily physical activity, while 51% reported daily fruit and vegetable consumption. Additionally, 22.6% had a history of elevated blood pressure, 8.2% reported previous elevated blood glucose levels, and 29.3% had a first-degree relative diagnosed with diabetes. Inadequate or problematic HL was reported in 59.4% of study participants. The median (IQR) sum score of DK, SE, and PHB was 23 (9), 51.7 (28.3), and 30 (14), respectively, indicating high DK and moderate SE and PHB. More than half of the participants (54.8%) were categorized as having moderate to high risk for T2DM (Table 1).

**Table 1:** Descriptive statistics of study participants (*N* = 416)

Variable		Category		*n* (%)	Median (IQR)
Participants’ demographics (as components of the ARABRISK)					
Age (years)		20-<30		33 (7.9)	41 (13)
		30-<40		147 (35.3)	
		40-<50		140 (33.7)	
		50–60		96 (23.1)	
Gender		Male		163 (39.2)	
		Female		253 (60.8)	
Education		Some high school or less		55 (13.2)	
		High school/diploma		289 (69.5)	
		Some university/or more		72 (17.3)	
**Other Demographics (not included in the ARABRISK)**					
Marital status		Married		391 (94%)	
		Single/widow/divorced		25 (6%)	
Monthly income (LE)		< 5000		176 (42.3)	
		5000-<10,000		157 (37.7)	
		≥ 10,000		83 (20)	
**Participants’ anthropometric**,** behavioral and medical data (as components of the ARABRISK)**					
Waist circumference (cm)	Female	< 80		72 (28.5)	84 (13)
		80–88		91 (36)	
		> 88		90 (35.5)	
	Male	< 94		76 (46.6)	94 (14)
		94–102		55 (33.7)	
		> 102		32 (19.7)	
BMI (kg/m^2^)		Normal (18.5–24.9)		90 (21.6)	28 (5.4)
		Overweight (25-29.9)		188 (45.2)	
		Obese I (30-34.9)		107 (25.7)	
		Obese II (≥ 35)		31 (7.5)	
Daily physical activity ≥ 30 min		Yes		106 (25.5)	
		No		310 (74.5)	
Daily consumption of fruits/vegetables		Yes		212 (51)	
		No		204 (49)	
Have ever had high blood pressure/have taken high blood pressure pills		Yes		94 (22.6)	
		No		322 (77.4)	
Have ever had high blood glucose		Yes		34 (8.2)	
		No		382 (91.8)	
Have ever given birth to a large baby (*n* = 161)		Yes		2 (1.2)	
		No		159 (98.8)	
Have first degree relatives ever been diagnosed with diabetes		Yes		122 (29.3)	
		No		294 (70.7)	
**Health literacy subscales**					
Health care (7)					5 (2)
Disease prevention (5)					4 (2)
Health promotion (4)					3 (1)
**Health literacy sum scale (16)**		Inadequate		75 (18)	12 (4)
		Problematic		172 (41.4)	
		Sufficient		169 (40.6)	
**Diabetes knowledge subscales**					
Risk factors (9)					6 (3)
Symptoms (10)					7 (2)
Complications (6)					4 (1)
Prevention (9)					5 (3)
**Diabetes knowledge sum scale (34)**					23 (9)
**Self-efficacy subscales**					
Healthy eating (100%)					54.5 (35.2)
Physical activity (100%)					50 (37.5)
**Self-efficacy sum scale (100%)**					51.7 (28.3)
**Preventive health behaviors subscales**					
Healthy eating (12)					8 (5)
Physical activity (12)					4 (3)
Weight management (12)					6 (4)
Smoking cessation/prevention (12)		Current smoker	77 (18.5)		12 (0)
		Quitted smoking	26 (6.3)		
		Non-smokers	313 (75.2)		
**Preventive health behaviors sum scale (48)**					30 (14)
**Diabetes Risk (ARABRISK score)**		Low		188 (45.2)	22 (13)
		Moderate		148 (35.6)	
		High		80 (19.2)	

Ethnicity was assigned a constant score of three for all participants based on ARABRISK criteria and thus is not reported separately

HL was significantly associated with higher DK, SE, and PHB and inversely associated with DR. The strongest correlation was observed between PHB and DR (a strong negative correlation), followed by moderate correlations between HL and PHB and between HL and DR. Detailed correlations are presented in Table 2.

**Table 2:** Correlations among study variables based on the hypothesized model

Variable	Health literacy	Diabetes knowledge	Self-efficacy	Preventive health behaviors
Diabetes knowledge (DK)	0.275 0.000*			
Self-efficacy (SE)	0.379 0.000*			
Preventive health behavior (PHB)	0.514 0.000*	0.223 0.000*	0.274 0.000*	
Diabetes risk (DR)	− 0.542 0.000*			− 0.957 0.000*

* Statistically significant at *p* < 0.001

Significant covariates associated with the exposure, mediators, and outcome variables, as identified through bivariate analysis (Additional file 1), were controlled for in the SEM analysis. The goodness-of-fit indices indicate that the hypothesized structural model linking HL to DR through DK, SE, and PHB provides a reasonable representation of the data. The GFI = 0.9773 and NFI = 0.9817 both exceed the recommended threshold of 0.90, suggesting a well-fitting model. Additionally, the SRMR = 0.0493 falls within the acceptable range (< 0.08), further supporting model adequacy. However, the RMSEA = 0.1298, 95% CI: 0.0847–0.1803 exceeds the conventional cutoff for a good fit, indicating that the model may benefit from refinements. Overall fit indices support the validity of the structural model, reinforcing the role of HL in influencing DR through DK, SE, and PHB (Table 3).

**Table 3:** Fit indices for the structural equation model assessing pathways between health literacy and diabetes risk

Fit index	Value
Chi-Square (x^2^)	23.9684
Degrees of Freedom (DF)	3
p-value for Chi-Square	0.000*
Root Mean Square Residual (RMR)	7.1192
Standardized RMR (SRMR)	0.0493
Goodness of Fit Index (GFI)	0.9773
Adjusted Goodness of Fit Index (AGFI)	0.8863
Root Mean Square Error of Approximation (RMSEA)	0.1298
RMSEA 90% CI (Lower-Upper)	0.0847–0.1803
Comparative Fit Index (CFI)	0.9839
Bentler-Bonett Normed Fit Index (NFI)	0.9817

*Statistically significant at *p* < 0.001

**Fig. 2:**
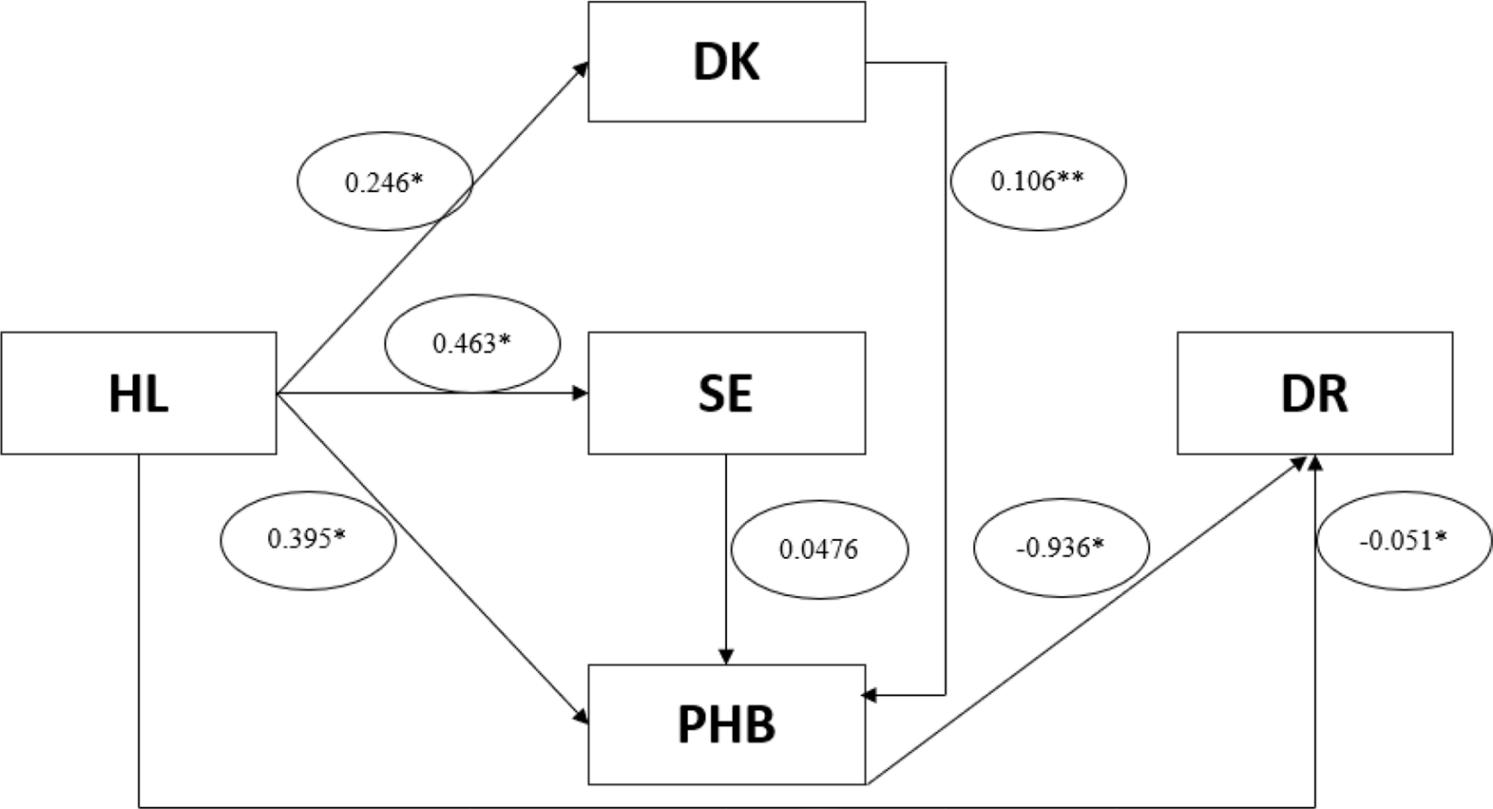
Pathway analysis model showing direct and indirect effects of health literacy on diabetes risk. *Statistically significant at *p* < 0.001; **statistically significant at *p* < 0.05. HL: Health literacy. DK: diabetes knowledge. SE: Self-efficacy. PHB: Preventive Health behaviors. DR: Diabetes risk

SEM analysis confirmed that HL had significant positive effects on DK, SE, and PHB, with a weak effect on DK and moderate effects on SE and PHB. The direct effect of HL on DR was very weak but statistically significant. The strongest association in the model was the negative effect of PHB on DR, which was very strong (β = − 0.93663, *p* < 0.001). The most substantial indirect effect of HL on DR occurred through PHB. Full model estimates are presented in (Fig. 2**)**.

## Discussion

This study examines how HL influences T2DM risk by validating a model that explores both direct and indirect pathways linking HL to DR scores in a non-diabetic population in Ismailia, Egypt. To the best of our knowledge, this is the first study analyzing the association between HL and DR using the ARABRISK score combined with the validated Arabic version of the HLQ-EU-16. Our study revealed that HL influenced DR both directly and indirectly, primarily through PHB. While the direct effect of HL on DR was statistically significant but weak, the strongest pathway was indirect — with HL increasing PHB, which in turn strongly reduced DR scores. Additional indirect effects were observed through DK and SE, though these were more modest.

The finding that over 50% of participants had a moderate to high risk for developing T2DM and that about 60% had problematic to inadequate HL, along with the moderate negative correlation between HL and DR scores (*r* = -0.542, *p* < 0.001), underscores HL as a key factor in diabetes prevention and supports the hypothesized direct pathway. This finding is consistent with research among non-diabetic adults in Germany, where lower self-reported HL was associated with a higher risk of T2DM, despite differences in assessment tools, such as the German Diabetes Risk Score, which does not account for gender differences [6]. Similarly, studies from Indonesia and Australia reinforce this association. The Indonesian study utilized the Finnish Diabetes Risk Score (FINDRISC), which closely aligns with the ARABRISK tool, except for the exclusion of the education item [33], while the Australian study applied the Australian Type 2 Diabetes Risk Assessment Tool, which includes smoking status but excludes BMI and education [8]. These findings collectively highlight the significance of HL in diabetes risk prediction across diverse populations and assessment methods.

Conversely, a recent study in Turkey found no significant correlation between HL scores and diabetes risk using the FINDRISK tool. The authors attributed this to the lack of sufficient diabetes-specific knowledge within general HL, suggesting that general HL alone may not adequately capture an individual’s understanding of DR or their likelihood of adopting PHB [34]. Additionally, they proposed that participants’ actual and perceived DRs were aligned, implying that even those with lower HL may still accurately assess their risk status, which could explain the absence of a correlation [35]. These findings underscore the importance of distinguishing between general and disease-specific HL and reinforce the need to investigate the pathways through which HL influences DR—particularly by examining DK as a potential mediator.

The strongest correlation observed in this study was between PHB and DR (*r* = -0.957, *p* < 0.001), surpassing both the direct correlation between HL and DR and all indirect pathways. This suggests that while cognitive factors such as HL and DK are essential for understanding and assessing risk**—**and SE plays a role in motivating behavior change**—**actual execution of PHB has the most direct impact on reducing DR. Although our study is cross-sectional and does not assess behavioral change over time, the strong PHB-diabetes risk correlation highlights the importance of PHB in diabetes prevention. This aligns with interventional studies showing that structured lifestyle modifications effectively reduce T2DM risk. For instance, a recent interventional study conducted among high-risk individuals in Thailand (an upper middle-income country) [36] found that participants in the intervention group had significantly higher PHB scores and lower blood sugar levels than the control group. The authors suggested these improvements were linked to healthier dietary habits, increased physical activity, and weight management, all contributing to reduced insulin resistance and improved insulin sensitivity.

Additionally, a systematic review and meta-analysis in low- and middle-income countries (LMICs), including Egypt (a lower middle-income country**)** [37], concluded that comprehensive lifestyle interventions are effective in preventing T2DM among at-risk populations. The review highlighted that these interventions contribute to DR reduction through improved insulin sensitivity, decreased inflammation, and weight loss, thereby preventing the transition from prediabetes to T2DM. While our study does not evaluate intervention effectiveness, these findings reinforce the plausibility of our results, suggesting that even in a non-interventional setting, lifestyle modifications play a critical role in DR reduction.

Numerous studies have established a significant relationship between HL and DK [15], SE [38], and self-care behaviors [39] in patients with diabetes. However, to the best of our knowledge, no study has examined these associations in a non-diabetic population. Therefore, direct comparisons with our findings are limited. Individuals living with diabetes typically engage with health services more frequently, receive disease-specific education, and develop behavioral routines based on their condition. These experiences likely strengthen the relationships between HL and DK, SE, and PHB. In contrast, non-diabetic individuals may have limited exposure to diabetes-related information or lack immediate motivation for behavior change, which can result in weaker or more indirect associations. The current study found a significant but weak positive correlation between HL and DK (*r* = 0.275, *p* < 0.001), suggesting that while higher HL contributes to greater DK, it is not the sole determinant. A meta-analysis of patients with T2DM by Marciano et al. [15] provides three key explanations for this weak correlation. First, performance-based HL assessments tend to show stronger correlations with DK than self-reported HL measures. Since our study used a self-reported HL tool, the observed association may underestimate the true HL-DK relationship. Second, general HL measures may not fully capture diabetes-specific literacy, as they assess overall health comprehension rather than knowledge directly relevant to diabetes risk and management. Third, most performance-based HL measures are not diabetes-specific, meaning that even when objective assessments are used, they may still fail to actually reflect an individual’s diabetes-related knowledge. These factors likely contributed to the weak HL-DK correlation observed in our study.

Similarly, this study found a significant but weak positive correlation between HL and SE (*r* = 0.379, *p* < 0.001), suggesting that while higher HL is linked to greater confidence in managing health, additional factors contribute to SE. A systematic review among diabetic patients supports this, indicating that HL is more strongly associated with SE when literacy skills—such as communicative and critical literacy—are considered, rather than basic functional literacy alone [40]. Since the tool used in our study assesses both general and communicative/critical literacy skills, this weak correlation suggests that other factors, beyond HL, may play a role in shaping SE. Additionally, the same review highlighted that SE is influenced by external factors such as social support, reinforcing the idea that interpersonal relationships and access to resources may significantly contribute to an individual’s confidence in managing their health.

In contrast, our study demonstrated a stronger correlation between HL and PHB (*r* = 0.514, *p* < 0.001), indicating that higher HL is associated with greater engagement in PHB. This aligns with previous research conducted on Iranian diabetic populations, which found that higher HL facilitates improved self-care behaviors through better access, understanding, and application of health information [39]. The stronger HL-PHB correlation, compared to HL-DK and HL-SE, suggests that HL has a more direct impact on PHB, while DK and SE may require additional influencing factors.

Additional evidence supporting the link between HL and PHB comes from a cross-sectional study conducted among urban adults in Iran, where Ranjbaran et al. (2022) [41] found a strong positive association between various HL dimensions and HPBs. These findings reinforce our results, highlighting that HL is not only a cognitive factor but also a practical enabler of behavior change. Although our population differs in DR status and setting, the consistent association between HL and PHB across diverse populations suggests that interventions aimed at improving HL may universally enhance PHB, thereby lowering chronic disease risks such as T2DM.

Furthermore, evidence from other health domains also supports the broader behavioral impact of HL. For instance, Rezakhani Moghaddam et al. (2022) [42] demonstrated that higher e-health literacy was significantly associated with the adoption of protective behaviors during the COVID-19 pandemic. Although this study focused on an acute infectious disease rather than a chronic condition, it highlights the same essential role of HL in guiding health behavior. Together, these studies suggest that HL—whether general or digital—is consistently associated with proactive health behavior across both routine, long-term disease prevention and urgent public health emergencies.

Findings from other health contexts further reinforce the consistent relationship between HL and preventive behaviors. For example, Fernandez et al. (2016) [43] found that among older adults in the U.S.A., higher HL—both self-reported and objective—was significantly associated with regular engagement in exercise, non-smoking behavior, and cancer screenings. Similarly, Darabi et al. (2025) [44] observed a strong positive relationship between HL and health-promoting lifestyle among patients with hypertension in Iran. Notably, in a study specifically addressing diabetes prevention, Abdi Almachavan (2024) [45] highlighted HL as a critical factor influencing the adoption of preventive health services and behaviors among individuals at risk for diabetes. Taken together, this growing body of evidence supports our study’s conclusion that HL is a modifiable determinant of PHB, with relevance across both diabetes-specific and broader chronic disease prevention contexts.

Furthermore, our study found weak but significant correlations between DK and PHB (*r* = 0.223, *p* < 0.001) and SE and PHB (*r* = 0.274, *p* < 0.001). This is consistent with findings from a study in a non-diabetic population in Singapore, which reported that PHB—particularly physical activity—was associated with better DK and stronger beliefs in diabetes prevention [46]. While both DK and SE contribute to PHB in our study, their influence appears modest compared to HL, which demonstrated a stronger direct correlation with PHB. This reinforces the idea that HL may be a more fundamental driver of PHB, shaping both knowledge acquisition and SE.

In the current study, the model fit indices suggest that the hypothesized pathways linking HL to diabetes risk through DK, SE, and PHB provide a reasonable representation of the data, reinforcing the study’s theoretical framework. While most indices indicate a well-fitting model, the slightly elevated RMSEA suggests that including additional covariates or alternative pathways may enhance model accuracy. The absence of directly comparable studies in a non-diabetic population highlights the novelty of this research and underscores the need for further validation across diverse populations.

In our study, the direct pathway “HL→ DR” was statistically significant but weak (β = -0.05108, *p* < 0.001), suggesting that HL alone may not be sufficient to substantially lower diabetes risk. While statistically significant—likely due to the adequate sample size and controlled modeling—this small effect size indicates a limited direct influence of HL on DR. Importantly, our SEM revealed that the influence of HL on DR is more pronounced through indirect pathways, particularly via PHB, which showed a very strong inverse association with DR. This finding aligns with a study conducted in a Chinese population, which found a similarly weak direct effect of HL on health status (β = 0.057), with SE and health behaviors acting as key mediators, reinforcing the role of indirect pathways [47]. Likewise, a meta-analysis on HL and diabetes self-management concluded that HL had a weak but significant effect on diabetes self-management variables, emphasizing that HL plays a role but requires additional factors for meaningful health improvements [48]. Furthermore, research suggests that HL interventions should integrate structured education and professional communication strategies to enhance understanding and engagement [49]. This is particularly relevant for individuals in early risk stages who may not yet perceive an urgent need for preventive actions, as HL alone may not be enough to drive behavior change unless combined with interventions that enhance motivation and risk perception.

As previously noted, the Turkish study using the FINDRISK tool reported no significant association between HL and DR [34]. Several methodological and contextual differences may explain the discrepancy in studies’ results. First, the ARABRISK tool includes an item on educational attainment, which is notably absent from the FINDRISK tool. Omission of such an item may reduce the sensitivity of FINDRISK to HL-related effects. Additionally, the scoring structures of the two tools differ substantially; FINDRISK assigns different weights and thresholds compared to ARABRISK, potentially influencing risk categorization and the detection of subtle associations. Second, the Turkish study relied on regression and correlation-based analyses, while our study used SEM, which enables simultaneous estimation of both direct and indirect effects. This approach may better capture the complex, mediated pathways through which HL influences DR. Lastly, differences in population characteristics, such as baseline HL levels, cultural norms, and health behavior patterns, may have contributed to the divergent findings. These comparisons highlight the importance of selecting appropriate risk assessment tools and using comprehensive analytical models when evaluating the impact of HL on chronic disease prevention.

The indirect pathway “HL → DK → PHB → DR” in our study revealed that HL had a moderate effect on DK, while DK had a weak effect on HB. This suggests that while knowledge contributes to health behavior engagement, it is not a standalone driver of behavior change. A path analysis in diabetic patients similarly found that DK alone does not guarantee improved HB unless reinforced by strong health beliefs, treatment adherence, and social support [50]. While this study focused on diabetic patients at risk of diabetic retinopathy, similar mechanisms may apply to non-diabetic individuals at risk of developing diabetes. Specifically, at-risk individuals—like those with diabetes—may require additional motivational factors beyond knowledge to translate awareness into sustained behavior change. Given that T2DM risk is often asymptomatic, individuals in early risk stages may not perceive an immediate need for lifestyle modifications, reinforcing the importance of interventions that enhance SE, risk perception, and behavioral reinforcement alongside knowledge acquisition.

The indirect pathway “HL → SE → PHB → DR” in our study showed that HL had a moderate positive effect on SE, aligning with findings from a systematic review in diabetic patients [40]. This reinforces the idea that HL enhances individuals’ confidence in managing their health, supporting its role in empowering SE across different populations. However, SE was not a significant predictor of PHB (β = 0.04768, *p* = 0.3327), suggesting that SE alone may not be a strong driver of behavioral change in this non-diabetic population. This contrasts with findings from a Korean study on diabetic patients with physical disabilities, which reported a significant indirect effect of HL on diabetes self-care behaviors through motivation and SE [51]. This discrepancy may be due to differences in study populations—while diabetic patients with established disease and disability may have a stronger sense of urgency and motivation to adopt self-care behaviors, individuals in our non-diabetic population may require additional factors, such as risk perception, external reinforcement, or structured interventions, to translate confidence into action.

Our study also showed a moderate significant effect of HL on PHB (β = 0.39576, p = < 0.001) in the indirect path “HL → PHB → DR.” This is consistent with findings from a previous study [38], which found a significant relationship between HL and self-care behaviors. The strongest indirect effect in this study was observed through the pathway “PHB→ DR,” with PHB showing the highest predictive value for DR reduction (β = -0.93663, p = < 0.001). This aligns with findings from a systematic review and meta-analysis, which demonstrated that adopting healthy behaviors can prevent progression to T2DM in individuals with impaired glucose tolerance or dysmetabolism [13]. Although our population was not diagnosed with these diseases, the results suggest that similar preventive mechanisms may apply, reinforcing the critical role of behavior modification in DR reduction.

This study has several strengths. It is among the first in Egypt—and the broader MENA region—to empirically examine both direct and indirect pathways linking HL to DR in a non-diabetic population using SEM. A key strength is the use of a comprehensive, theory-driven model that incorporates multiple mediators to explore the complex mechanisms through which HL may influence DR. The application of validated and culturally adapted tools further enhances the study’s reliability and contextual relevance. Additionally, the relatively large and randomly selected sample increases internal validity and provides valuable insight into an at-risk but often under-studied population.

This study is not without limitations. First, its cross-sectional design prevents causal inference, limiting the ability to determine whether HL directly influences diabetes risk over time. Second, reliance on self-reported HL, DK, SE, and PHB may introduce reporting bias, potentially affecting the accuracy of associations. Third, the study was conducted among university employees, which may limit the generalizability of findings to the broader population. Fourth, the HL tool used assessed general HL with communicative and critical literacy but did not specifically measure diabetes-related HL, which may have influenced the observed associations. Fifth, diabetes risk, as the outcome measure, was based on a risk score rather than an objective clinical assessment. Additionally, while several confounders were adjusted for, other potential factors, such as psychological influences, were not assessed.

## Conclusion

A large proportion of our study participants had limited HL, and over half were at moderate to high risk of developing type T2DM. Health literacy was positively associated with DK, SE, and PHB and negatively associated with DR. Among these, PHB had the strongest protective effect. In path analysis, while HL had a weak but significant direct effect on DR, its impact was more substantial through indirect pathways involving DK, SE, and PHB. The strongest influence was through PHB, highlighting their central role in reducing diabetes risk. These findings carry practical implications for public health and diabetes prevention strategies. Interventions that focus on improving HL— especially those that empower individuals to adopt and maintain preventive health behaviors—could be a cost-effective approach to reducing diabetes risk among at-risk, non-diabetic populations. Tailoring educational and behavioral programs to address HL gaps in workplaces or communities may help identify individuals at early risk and support them in making informed health decisions, ultimately contributing to lower disease burden and reduced strain on healthcare systems.

Future research should build on these findings using longitudinal or intervention-based study designs to assess how improvements in HL affect DR over time. Incorporating diabetes-specific HL tools and objective behavioral and clinical measures would enhance validity. Furthermore, exploring additional psychosocial mediators such as perceived risk, motivation, or social support could deepen understanding of the mechanisms through which HL influences preventive behaviors. Finally, expanding this research to include diverse populations across different settings in Egypt could inform broader, context-specific public health strategies.

## Electronic supplementary material

Below is the link to the electronic supplementary material.

Supplementary Material 1

## Data Availability

No datasets were generated or analysed during the current study.

## Abbreviations

ADA: American Diabetes Association
BMI: Body Mass Index
CFI: Comparative Fit Index
CI: Confidence intervals
CP: Comparative prevalence
DK: Diabetes knowledge
DM: Diabetes Mellitus
DR: Diabetes risk
DM: Diabetes Mellitus
GFI: Goodness of Fit Index
HB: Health behaviors
HL: Health literacy
IDF: International Diabetes Federation
IQR: Interquartile range
MENA: Middle East and North Africa
NFI: Normed Fit Index
PHB: Preventive health behaviors
RMSEA: Root Mean Square Error of Approximation
SEM: Structural Equation Modeling
SRMR: Standardized Root Mean Square Residual
T2DM: Type 2 Diabetes Mellitus
WC: Waist circumference
WHO: World Health Organization
