# Supplementary material for: Pathways linking health literacy to diabetes risk scores in a non-diabetic population in Ismailia, Egypt: a cross sectional study design
Source: BMC Public Health. 2025 Jun 25;25:2170. doi: 10.1186/s12889-025-23526-1 (PMC12188667; doi:10.1186/s12889-025-23526-1)
Supplement: Supplementary file 1 — Supplementary Material 1 [file 12889_2025_23526_MOESM1_ESM.docx]

**Table 1** Bivariate analysis of covariates associated with the exposure, mediators, and outcome variable among study participants

| **Covariates** | **Health literacy** | **Diabetes knowledge** | **Self-efficacy** | **Preventive health behaviors** | **Diabetes risk**^a^ |
| --- | --- | --- | --- | --- | --- |
| **Age**^b^ | -.314  0.000*** | -.054  .273 | -.286  0 .000*** | -.583  0.000*** |  |
| **Gender**^c^ | 0.390 | 0.025* | 0.208 | 0.001** |  |
| **Education**^d^ | 0.000*** | 0.005** | 0.007** | 0.000*** |  |
| **Marital status**^c^ | 0.453 | 0.155 | 0.103 | 0.564 | 0.629 |
| **Income**^c^ | 0.000*** | 0.002** | 0.005** | 0.000*** | 0.000*** |
| **Waist circumference**^b^ | -.101  0.040* | -.128  0.009** | -.048  0.334 | -.378  0.000*** |  |
| **Body mass index**^b^ | -.201  0.000*** | -.064  0.195 | -.123  0.012* | -.450  0.000*** |  |
| **Have ever had high blood glucose**^c^ | 0.008** | 0.851 | 0.251 | 0.000*** |  |
| **Have ever had high blood pressure/have taken high blood pressure pills**^c^ | 0.000*** | 0.225 | 0.006** | 0.000*** |  |
| **Have first degree relatives ever been diagnosed with diabetes**^c^ | 0.003** | 0.503 | 0.305 | 0.000*** |  |
| **Smoking status**^d^ | 0.011* | 0.061 | 0.070 | 0.000*** | 0.000*** |

*Statistically significant at the 0.05 level (2-tailed); **statistically significant at p < 0.01; *** Statistically significant at p < 0.001

^a^Not all covariates were examined in relation to diabetes risk, as some of these variables are components of the ARABRISK tool used to measure diabetes risk.

^b^Analysis performed using Spearman’s Rank Correlation

^c^Analysis performed using Mann-Whitney U test

^d^Analysis performed using Kruskal-Wallis test
